# Supplementary material for: A parametric framework for multidimensional linear measurement error regression
Source: PLoS One. 2022 Jan 21;17(1):e0262148. doi: 10.1371/journal.pone.0262148 (PMC8782541; doi:10.1371/journal.pone.0262148)
Supplement: Attachment — Submitted filename: ResponseToReviewers.pdf [file pone.0262148.s001.pdf]

PONE-D-21-22997

A parametric framework for multidimensional linear measurement error regression

Stanley Luck

General remarks

The line numbers correspond to the 'tracked changes' document.

- In accordance with the reviewers comments, there are several changes that have significantly improved the paper. I thank the editor and the reviewers for their careful review and giving me the opportunity to revise the manuscript.
- The paper has a new title. The term "measurement error" refers to a sub-discipline of statistics with an extensive research literature and a long history. Some familiarity with MER research is needed for assessing the 'novelty' of this work.
- This paper on LRVVs is the third in a series discussing my work on the *applied algebraic foundations* of data analysis (Luck 2019, Luck 2020). The Introduction already states that the objective of this work is the investigation of linear "measurement error regression (MER) methods for *multivariate* data" (line 26). In this work, I show that fitting a multidimensional line to LRVV data is an important pedagogical problem for establishing the principles of multivariate data analysis. This includes the formulation of slope, covariance and correlation in a more general way for the parametric representation.

- The OLR method requires that data for the $\mathbf{x}$ variable be error free, a condition that is rarely obtained in experimental data. Therefore, the OLR algorithm is applicable to a limited class of regression analysis problems. Then, a statistics student might conclude that the textbook treatment of linear regression is incomplete. The parametric linear regression (PLR) framework is more general because it takes into account the experimental error in all variables, and includes OLR as a special case.

- There are longstanding problems and controversies in statistics as demonstrated by the wide range of opinions among statisticians on the merits of p-value and statistical significance (Wasserstein 2019). This lack of consensus on the merits of various widely used statistical measures (see line 6-17) is detrimental for statistics teaching and practice. This includes the treatment of measurement error in linear regression, covariance, and the attenuation of Pearson correlation coefficient. These unresolved statistical problems partly explain why there is a reproducibility crisis in science (NAS 2019). The satisfactory resolution of these controversies is important for progress in many areas of research. The investigation of these problems was an integral part of my previous work at DuPont.

- Finally, I note that the experimental data for this paper are publicly available.

Luck, Stanley. 2019. "Factoring a 2 x 2 Contingency Table." Edited by Fabio Rapallo. PLOS ONE 14 (10): e0224460. https://doi.org/10.1371/journal.pone.0224460.

Luck, Stanley. 2020. "Nonoverlap Proportion and the Representation of Point-Biserial Variation." Edited by Alan D Hutson. PLOS ONE 15 (12): e0244517. https://doi.org/10.1371/journal.pone.0244517.

National Academies of Sciences, Engineering, and Medicine. 2019. Reproducibility and Replicability in Science. Washington, D.C.: National Academies

Press. https://doi.org/10.17226/25303.

Wasserstein, Ronald L., Allen L. Schirm, and Nicole A. Lazar. 2019. "Moving to a World Beyond 'p < 0.05.'" The American Statistician 73 (sup1): 1–19. https://doi.org/10.1080/00031305.2019.1583913.

List of key changes

- Line 56 - 70. The list of main novel contributions has been amended to include the corresponding equation numbers and propositions.
- Fig 3 now includes a CVE curve for Deming weighting.
- Line 342. Remark on Pearson correlation tensors and two-way, three-way, ..., m-way weighted averages, etc.
- Line 423. Section 1.7 in the original manuscript has been moved to the 'Data analysis and results' section.
- The Discussion has been revised.

Response to reviewers

My responses to the reviewers comments are highlighted in blue.

0.1 Reviewer #1

In this paper, the author proposed "The chain rule, measurement error regression and RNA-Seq analysis".

The strengths of the paper are that it is well structured, the description of the related work is well done and that results are extensively compared to results of the similar research.

1) Draw a graphical abstract of this work.

A 'graphical abstract' has been created by amending the list of the main novel contributions (line 56-70) to include the corresponding equation numbers and propositions. PLOS ONE rules for the placement of figures do not allow me to include the figure numbers.

(Fig 3; Eq 13,16,17), (Fig 1A,1B,1D,1E,2A; Eq 22,23; Proposition 1), (Fig 1F; Eq 6,7), (Fig 1C,2B; Eq 32,33,35), (Fig 5,6; Eq 40,43).

2) Justify the novelty of the proposed approach?

In this work, I discuss the fact that statistical measures of linear dependence, including covariance, correlation coefficient and regression slope are subject to the chain rule. This is a novel idea with broad implications for multivariate statistics and data science. The paper already includes references to many important MER papers that serve as a starting point for assessing the 'novelty' of this work. I find that the statistical quantities and expressions that have not been previously reported in the scientific literature include Eq 6, 7, 13, 16, 17, 22, 23, 25, 27, 32, 33, 35, 37, and 43.

3) Proofread the article once again?

Done.

4) The experimental validation of the proposed approach is quite confusing justify it.

- 'Experimental validation' is not required for the OLR and Moore-Penrose pseudo-inverse algorithms because they are constructed from algebraic least-squares principles (Noble 1977; Keener 1988; Boyd 2018).
- PLR is algebraically constructed by extending the standard least-squares regression framework with the chain rule to transform to the parametric representation. Therefore, the validation is determined *a priori* by

whether the PLR framework is consistent with underlying least-squares theory for overdetermined systems of equations. Therefore, the PLR algorithm does not require an 'experimental validation'.

- However, our discussion of PLR includes graphs that serve as visual illustrations of statistical concepts to make the paper more accessible. The graphs are obtained from Monte Carlo simulations of MER and examples of PLR using publicly available data.

Boyd, Stephen, and Lieven Vandenberghe. 2018. Introduction to Applied Linear Algebra. Cambridge University Press. https://doi.org/10.1017/9781108583664.

Keener, James. 1988. Principles Of Applied Mathematics: Transformation And Approximation -. New York: Addison-Wesley Pub. Co.

Noble, Ben, and James W. Daniel. 1977. Applied Linear Algebra. 2nd ed. Englewood Cliffs: Prentice-Hall.

5) Give some experimental comparison of the proposed approach with existing approach?

For bivariate data, Fig 1D shows the experimental comparison of PLR with existing approaches. For multivariate data such those shown in Fig 5, it is not possible to provide an experimental comparison because there are no alternative methods for fitting a multidimensional line. There is no previously published work on the multidimensional linear regression problem.

0.2 Reviewer #2

The manuscript reading looks more like a technical report (heavy on the methodological description).

See my response to question #0.1-4 above. This paper describes novel PLR methodology which has broad applications in data analytics. The mathematical emphasis is intentional because the goal is to identify principles for multivariate

data analysis.

To attract strong interest, it is highly recommended to revise the manuscript to focus on the major objects and findings in a concise manner.

The major 'objects and findings' are already summarized in the Introduction, see my response to question #0.1-1 above. See lines 26, 51, 75, 164, and 202 for statements about the objectives of this work.

I have also some comments which are given below.

1. Title: Consider modifying it to be more specific. The wording of this current title (”The chain rule, measurement error regression and RNA-Seq analysis”) is somewhat imprecise.

The paper has a new title.

2. In page no 3 and 4 author mentioned seven novel contributions of his work but they are not clearly established in the whole paper.

See my responses to questions #0.1-1 and #0.1-2 above.

3. Recommendation to revise the “data analysis and results” part in details.

The “data analysis and results” section has been revised.

4. The discussion part (page no. 23 and 24) does not clearly state the point of view of this paper. The revision is needed for this part.

The Discussion has been revised.

5. I recommend that the author should revise the Fig. 1, Fig. 5 and Fig. 6 to illustrate the workflow. Assist the reader by providing some intuition.

The discussion of RNA-Seq data analysis is now entirely contained in the 'Data analysis and results' section. The PLOS ONE rules for the placement of figures prevents me from making a figure in the 'Data analysis' section to contain Fig 1D, E, F.

6. PLOS authors have the option to publish the peer review history of their article (what does this mean?). If published, this will include your full peer

review and any attached files.

This ‘response to reviewers’ document will be published as-is if the paper is accepted for publication.
